# Mimicry and mitonuclear discordance in nudibranchs: New insights from exon capture phylogenomics

**DOI:** 10.1002/ece3.6727

**Published:** 2020-09-17

**Authors:** Kara K. S. Layton, Jose I. Carvajal, Nerida G. Wilson

**Affiliations:** ^1^ Centre for Evolutionary Biology School of Biological Sciences University of Western Australia Crawley WA Australia; ^2^ Collections & Research Western Australian Museum Welshpool WA Australia; ^3^ School of Biological Sciences, Zoology Building University of Aberdeen Aberdeen UK

**Keywords:** exon capture, mimicry, mitonuclear discordance, Nudibranchia, phylogenomics, speciation

## Abstract

Phylogenetic inference and species delimitation can be challenging in taxonomic groups that have recently radiated and where introgression produces conflicting gene trees, especially when species delimitation has traditionally relied on mitochondrial data and color pattern. *Chromodoris*, a genus of colorful and toxic nudibranch in the Indo‐Pacific, has been shown to have extraordinary cryptic diversity and mimicry, and has recently radiated, ultimately complicating species delimitation. In these cases, additional genome‐wide data can help improve phylogenetic resolution and provide important insights about evolutionary history. Here, we employ a transcriptome‐based exon capture approach to resolve *Chromodoris* phylogeny with data from 2,925 exons and 1,630 genes, derived from 15 nudibranch transcriptomes. We show that some previously identified mimics instead show mitonuclear discordance, likely deriving from introgression or mitochondrial capture, but we confirm one “pure” mimic in Western Australia. Sister–species relationships and species‐level entities were recovered with high support in both concatenated maximum likelihood (ML) and summary coalescent phylogenies, but the ML topologies were highly variable while the coalescent topologies were consistent across datasets. Our work also demonstrates the broad phylogenetic utility of 149 genes that were previously identified from eupulmonate gastropods. This study is one of the first to (a) demonstrate the efficacy of exon capture for recovering relationships among recently radiated invertebrate taxa, (b) employ genome‐wide nuclear markers to test mimicry hypotheses in nudibranchs and (c) provide evidence for introgression and mitochondrial capture in nudibranchs.

## Graphical abstract

We employ exon capture to resolve the phylogeny of a recently radiated genus of toxic nudibranch and to test previous mimicry hypotheses. We uncover mitonuclear discordance for the first time in nudibranchs that derives from mitochondrial capture or introgression, and we confirm one mimetic species in Western Australia. This study advances understanding of nudibranch diversity and evolution and it demonstrates the efficacy of exon capture for resolving phylogenetic relationships in recently radiated taxa.

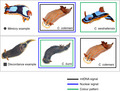

## 1 INTRODUCTION

Delimiting species boundaries and defining phylogenetic relationships are essential to our understanding of evolution (Soltis & Soltis, 2000), but resolving these relationships can be difficult when lineages have recently radiated (Giarla & Esselstyn, 2015) and where introgression further complicates phylogenetic inference (Harrington, Benavides, & Near, 2013). This work is especially problematic in taxonomic groups where species delimitation has traditionally relied on mitochondrial data and color pattern because introgression is undetectable without information from the nuclear genome and it can facilitate the evolution of mimicry (Enciso‐Romero et al., 2017). This is particularly true for tropical nudibranchs, where systematic relationships remain problematic due to poor resolution, high levels of diversity, and the recent discovery of mimicry in some groups (Layton, Gosliner, & Wilson, 2018; Padula et al., 2016). Although mimicry has been extensively studied in many terrestrial taxa (e.g., butterflies, Flanagan et al., 2004; *Heliconius* Genome Consortium, 2012; millipedes, Marek & Bond, 2009; and frogs, Chouteau, Summers, Morales, & Angers, 2011), less is known about mimicry in marine invertebrates.

Chromodorid nudibranchs have evolved to selectively sequester toxic chemicals from their sponge prey and many display aposematic signals (e.g., Cimino & Ghiselin, 1999; Rudman, 1991). Previous work has identified a Müllerian mimicry ring in eastern Australia, where multiple chemically defended *Chromodoris* species appear very similar (Cheney et al., 2016), and a second quasi‐Batesian system in chromodorids where some species are unpalatable but non‐toxic (Winters, White, et al., 2018). The type genus, *Chromodoris*, is brightly colored and exhibits blue, black, yellow, and white color patterns, similar to the warning colors found in other mimetic taxa (e.g., *Heliconius*, Mallet, 2010). Müllerian mimicry can evolve by mutation or through introgression among species, the latter of which has been well documented in butterflies (Enciso‐Romero et al., 2017; Pardo‐Diaz et al.,^2012^), but identifying the origin of mimicry is difficult for groups lacking genomic resources. Previous studies have relied onmitochondrial data for phylogenetic reconstruction in *Chromodoris*, which prevents the detection of introgression, and the resulting phylogenies were poorly resolved with short branch lengths, indicating that a recent radiation has occurred (Johnson & Gosliner, 2012; Layton et al., 2018; Turner & Wilson, 2008; Wilson & Lee, 2005). Layton et al. (2018) also identified up to four distinct color patterns associated with a single mitochondrial clade that matched other congenerics, indicating the presence of mimicry in this genus. Given the lack of resolution in the existing *Chromodoris* phylogeny, and the evidence for mimicry and a recent radiation, new methods are needed to clarify evolutionary relationships and patterns in this complex genus.

Next‐generation sequencing techniques produce large amounts of genome‐wide data that can be used to improve phylogenetic resolution (e.g., Hackett et al.,^2008^; McCormack et al.,^2013^) and reduced representation techniques that target‐specific sections of the genome are becoming increasingly popular for this work. Exon capture, which utilizes transcriptomes to establish a universal set of loci that can be targeted with probes, has been employed for phylogenetic reconstruction in several taxonomic groups (e.g., Bi et al.,^2012^; Bragg, Potter, Bi, & Moritz, 2016; Hugall, O’Hara, Hunjan, Nilsen, & Mousalli, 2016; Quattrini et al.,^2018^; Teasdale, Kohler, Murray, O’Hara, & Moussalli, 2016), but its utility for resolving phylogenetic relationships at shallow scales of divergence and in cases where species delimitation is problematic has rarely been tested.

Our objectives in this study were to (a) assess the efficacy of exon capture for resolving phylogenetic relationships in a group of recently radiated nudibranchs, (b) test hypotheses of mimicry in the genus, and (c) assess topological variability among different gene sets and phylogenetic methods.

## 2 METHODS

### 2.1 Sample collection in RNAlater

Two *Chromodoris* (*C. magnifica*, *C. westraliensis*), ten chromodorids from other genera, and one *Actinocyclus* were collected by hand using SCUBA at depths of 7–17 m from sites in San Diego (California), Mooloolaba (Queensland), Port Philip Bay (Victoria), Exmouth Gulf (Western Australia), and Perth (Western Australia) between 2015 and 2017. Samples of foot tissue from each specimen were finely diced with excess mucus removed before being placed in RNAlater at 4°C for 24hr and then transferred to −80°C for storage. Whole voucher specimens were preserved in 100% ethanol.

### 2.2 RNA extraction, transcriptome sequencing, and de novo assembly

Total RNA was extracted from RNAlater samples using either the QiagenRNeasy kit following manufacturer's protocols or by homogenizing in TRIzol reagent for 1min with zirconium oxide beads and subsequently following manufacturer's protocols for the Zymo Direct‐zol purification kits including the optional DNAse step. The quantity of RNA was determined with Nanodrop, Qubit and gel visualization. RNA purity was quantified using Nanodrop 260/280 nm absorption ratios with ratios close to 2.0 considered pure RNA and a volume of 40μl was used for multiplexed sequencing. Transcriptomes were sequenced on an Illumina HiSeq 2000 platform, pooling 5–6 libraries per lane, using 100bp paired end reads by the Australian Genome Research Facility (Melbourne). In order to enhance computational efficiency, a random subset of 20,000,000 paired reads were selected from the four transcriptomes with more than 20M reads (*Actinocyclus verrucosus*, *Chromodoris magnifica*, *Felimida macfarlandi*, *Verconia norba*) prior to assembly. Randomly subsampling reads did not affect downstream analysis because rare transcripts were not assessed in this study. Two additional nudibranch transcriptomes (*Prodoris clavigera*, *Doris kerguelenensis*) were downloaded from GenBank. Both the subsetted transcriptomes (*N* = 4) and the full transcriptomes (*N* = 11) were assembled de novo using the transcriptome pipeline in Agalma (Dunn, Howison, & Zapata, 2013) employing default parameters and a minimum read quality threshold of 32. Transcripts with <0.03 Fragments per Kilobase Million (FPKM) were removed after assembly to remove weakly expressed isoforms of transcripts or incorrectly identified isomers due to sequencing errors.

### 2.3 Identifying single copy homologous genes

**Figure 1:**
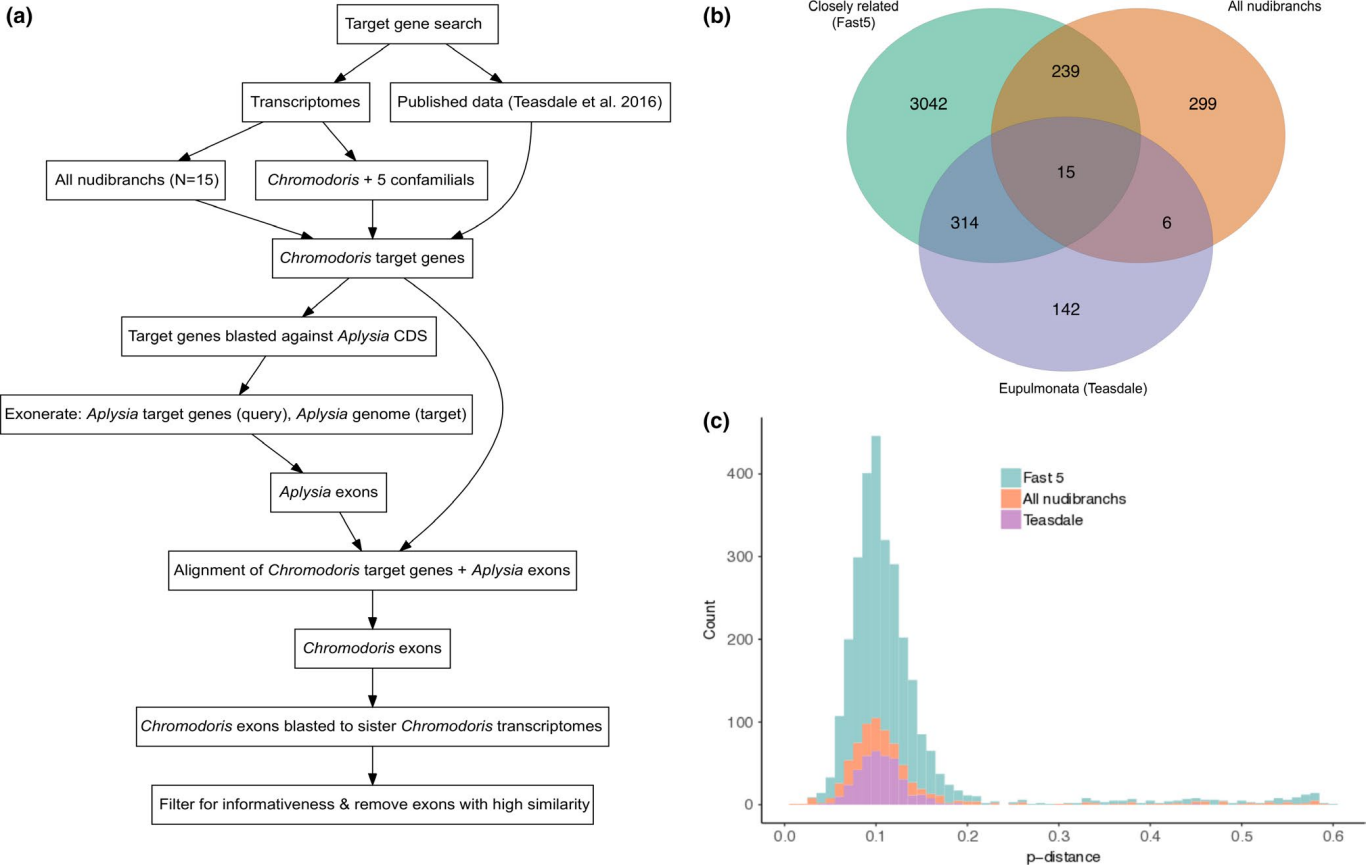
Gene search. (a) Workflow outlining steps for target gene search and identifying Chromodoris exon sequences. (b) Homologous genes retrieved from two Agalma searches in this study (Fast 5, All nudibranchs) and a published dataset in Teasdale et al. (2016). (c) Pairwise distance of gene alignments of *Chromodoris westraliensis* and *Goniobranchus coi* from two Agalma searches (All nudibranchs amino acid, Fast 5 nucleotide) and the Teasdale et al. (2016) dataset

A maximum‐likelihood phylogeny of 1,126 genes from all transcriptomes (see “all nudibranchs” below) was constructed using a single partition and a GTR20 + F substitution model in IQtree v1.6.8 (Nguyen, Schmidt, von Haeseler, & Minh, 2015). This transcriptome phylogeny was used to construct a framework for interpreting gene and taxon selection in downstream analyses. Multiple methods of target gene identification were used to identify, compare, and maximize the number of phylogenetically informative genes for *Chromodoris*. A workflow for target gene search and identifying exon sequences is available in Figure 1a. These were identified from three comparisons across different taxonomic levels; (a) between *Chromodoris* and closely related genera (five transcriptomes; “Fast 5”), (b) across the family Chromodorididae and other nudibranch outgroups (fifteen transcriptomes; “all nudibranchs”), and (c) across eupulmonate gastropods (Teasdale et al., 2016). Single copy homologous genes were identified from two different searches using the phylogeny pipeline in Agalma. The first set of homologous genes was recovered with a nucleotide search of five closely related species (*C. magnifica*, *C. westraliensis*, *Doriprismatica atromarginata*, *Goniobranchus coi*, *G. fidelis*). The second set of homologous genes was then recovered with an amino acid search of all fifteen nudibranch transcriptomes, and data were extracted from the Agalma output using the matrix2genes function. The Agalma phylogeny pipeline selects genes that are phylogenetically informative and produce gene trees with no more than one terminal per taxa (i.e., paralogy pruning). The motivation for conducting these two searches was to target genes that would be informative for resolving both deep and shallow‐scale evolutionary relationships. We calculated pairwise distances in Geneious v9.0 (Kearse et al., 2012) between the two most complete transcriptomes (*C. westraliensis*, *G. coi*) in order to evaluate the distribution of evolutionary rates between the results from the two Agalma searches since we predicted that these gene sets would resolve relationships at different evolutionary scales. Finally, a third set of target genes was taken from Teasdale et al. (2016) who identified a set of 500 genes from eupulmonate gastropods that were predicted to have broad phylogenetic utility.

### 2.4 Identifying exon/intron boundaries

The *Lottia gigantea* gene IDs listed in Teasdale et al. (2016) were downloaded from Genbank and combined with the two sets of target genes identified with the Agalma v1.0 pipeline to generate a single set of genes. These targets were then blasted against the *Aplysia californica* CDS genome to identify corresponding genes in *Aplysia*. The *A. californica* genome was selected as a reference because it was the best‐assembled genome in Heterobranchia, the subclass that Nudibranchia belongs in. We then used Exonerate v2.2.0 (Slater & Birney, 2005) to find introns by specifying the *Aplysia* genes as the query and the *Aplysia* genome (AplCal2.0 assembly Broad Institute) as the target. The output from Exonerate, a text file with concatenated exons per gene, was parsed into individual exons and exons >200 bp were saved using a custom python script. To find the corresponding exons in the *Chromodoris* transcriptomes, Exonerate was run again using these *Aplysia* exons as the query and the *C. magnifica* and *C. westraliensis* transcriptomes as targets. Exons were discarded from the Exonerate output if they were less than 115 bp in length, less than 65% similar to the *Aplysia* query exons, or longer than the original query exons. The single best sequence for each exon from either *C. magnifica* or *C. westraliensis* was selected based on highest score and all resulting exons were combined into a single file. These “best” exons were reciprocally blasted against each *Chromodoris* transcriptome to select genes that were variable (and thus informative) between the two *Chromodoris* species. A custom python script was then used to filter the results of the reciprocal blast based on percent identity. Only BLAST hits that were 92%–99% similar were retained, as hits that were 100% similar would not be informative within *Chromodoris* and hits <92% similar may have been paralogs. If an exon blasted to any other exon in the target set, both were removed from the target set to prevent baits from hybridizing to multiple targets. Sequences of target exons from *C. westraliensis* were sent to Arbor Biosciences (formerly MYcroarray) in Ann Arbor, Michigan for bait design and synthesis.

### 2.5 DNA extraction and targeted sequencing

The majority of DNA extractions for exon capture were taken from previous work by Layton et al. (2018) using samples of foot tissue from 65 *Chromodoris* specimens preserved in 96% ethanol, and an additional specimen preserved in 4% glutaraldehyde. Four additional DNA extractions were performed in this study for outgroup taxa (*Ardeadoris egretta*, *D. atromarginata*, *G. coi*, *G. fidelis*) using Qiagen DNeasy blood and tissue kits with an elution volume of 100–200 μl following manufacturer's protocols. Extractions were quantified on a Qubit, and in cases where concentrations were low, several extractions were prepared from the same animal and combined and concentrated using a Zymo DNA Clean and Concentrator kit following manufacturer's protocols. A total of 69 samples were sent to Arbor Biosciences for library preparation and target capture using the MYbaits‐1‐12 targeted sequencing kit protocols. DNA samples were sheared with sonication using a Qsonica Q800R instrument to an average insert length of 250bp, and eight libraries were pooled per reaction for a total of nine multiplex capture reactions. Sequencing was performed on a half lane of the Illumina HiSeq 2,500 platform with 100bp paired end reads also at Arbor Biosciences.

### 2.6 Bioinformatic processing, phylogenetic analysis, and SNP calling

Trimmomatic v0.36 (Bolger, Lohse, & Usadel, 2014) was used to remove adapter sequences, exon capture reads with a quality score below 15 in a 4‐bp sliding window, and reads shorter than 26 bp. HybPiper v1.3.1 (Johnson et al., 2016) was employed to assemble the cleaned reads into contigs of the targeted regions of the genes. Briefly, the reads were mapped to a reference file of concatenated bait sequences using Burrows‐Wheeler Aligner (BWA) (Li & Durbin, 2009). Mapped reads were then assembled de novo by gene using SPAdes v3.13.0 (Bankevich et al., 2012), and the resulting contigs were trimmed to include only the targeted exons with Exonerate v2.2.0. HybPiper produced an unaligned fasta file for each gene, containing a DNA sequence for each sample, and a series of summary statistics. For example, Hybpiper uses BWA to map the reads to contigs to present a value for percent reads on target. Genes that did not enrich or enriched poorly (genes whose contigs were <50% of the reference) were removed (*n* = 125). The resulting gene files were aligned using MAFFT v7 (Katoh & Standley, 2013). All gene alignments were trimmed with the “strict” method in trimAl v1.2 (Capella‐Gutiérrez, Silla‐Martinez, & Gabaldon, 2009) after conducting a series of tests to compare the “gappyout” and “strict” options. A total of 1,630 gene alignments were then used for phylogenetic analysis. The number of parsimony‐informative sites in the final alignment was calculated in Geneious v9.0 (Kearse et al., 2012).

Phylogenetic analyses were conducted separately on two different datasets; (a) the full dataset containing all loci identified in this study, including loci identified by Teasdale et al. (2016), and (b) only those loci identified by Teasdale et al. (2016). We chose to assess the informativeness of just those loci identified by Teasdale et al. (2016) as these were predicted to have broad phylogenetic utility across Gastropoda. A mitochondrial (COI, 16S) dataset derived from Layton et al. (2018) was also used for comparing tree landscapes in the Treespace package in R (Jombart, Kendall, Almagro‐Garcia, & Colijn, 2017). Individual gene alignments were concatenated for model testing (Kalyaanamoorthy, Minh, Wong, von Haeseler, & Jermiin, 2017) and ML analysis in IQtree v1.6.8 (Nguyen et al., 2015) with 1,000 ultrafast bootstrap replicates (Hoang, Chernomor, von Haeseler, Minh, & Vinh, 2018). Nodes with <50% bootstrap support were collapsed in the ML phylogeny. Additionally, individual gene ML trees were constructed with the same methods and then used as an input for summary coalescence analysis in ASTRAL II (Mirarab & Warnow, 2015) with 100 bootstrap replicates. The cophylo function in the Phytools package in R (Revell, 2012) was used to examine congruence in multiple sets of trees. Bootstrap trees and consensus trees for each exon dataset (i.e., full gene, Teasdale gene) and each tree‐building method (i.e., concatenated ML, ASTRAL) were used to explore topological variability in the Treespace package in R using the Kendall Colijn distance metric to produce the PCA (Jombart et al., 2017). For the mtDNA dataset, bootstrap and consensus trees from an ML analysis in Layton et al. (2018) were explored in Treespace. The diversity of phylogenetic tools and genetic datasets employed in this study provided a unique opportunity for comparing topological variability among methodologies.

HybPiper produced a binary alignment map (BAM) file for each sample, which was used to call variants with the Genome Analysis Tool Kit v4 (GATK) (McKenna et al., 2010) following the GATK best practices for variant calling. PCR duplicates were removed from the BAM files which were then sorted and used to call SNP variants using GATK haplotype caller in gVCF mode. Variants were selected for analysis after a hard filtering step (QD < 2.0, MQ < 40.0, FS > 60.0, SOR > 3.0, MQRankSum < −12.5, ReadPosRankSum < −8.0) using the GATK SelectVariants tool. The resulting VCF file was pruned for linkage disequilibrium in PLINK 2.0 (Chang et al., 2015) by removing SNPs that were correlated (*r* ^2^ > 0.2) to any other SNP in a 50bp sliding window. The pruned VCF file was used with a custom python script to find alleles that were fixed differently in putative parental lineages for individuals where hybridization was suspected. We report this as the percentage of heterozygous fixed alleles. In F1 or F2 hybrids, we would expect the heterozygosity rate of alleles that are fixed differently between the two parental lineages to be very high. To determine whether a signal of introgression was present among closely related species, we ran hybridization detection using phylogenetic invariants (HyDe) (Blischak, Chifman, Wolfe, & Kubtako, 2018) with the concatenated exon alignment and all individuals. We ran the full hybridization detection analysis using the *run_hyde.py* script to test all possible triplet combinations (Blischak et al., 2018).

## 3 RESULTS

### 3.1 Searching for homologous loci in nudibranchs

**Figure 2:**
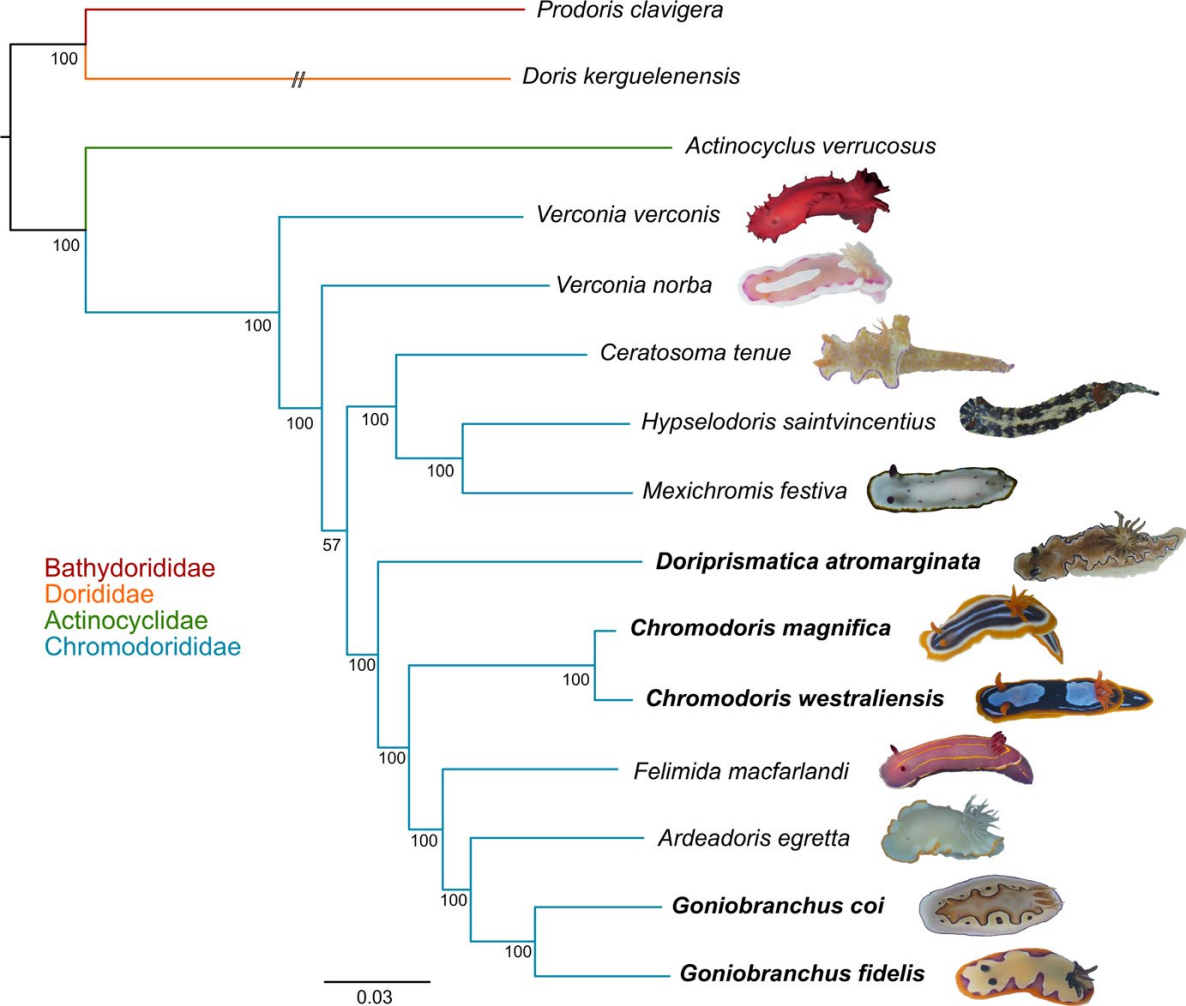
Transcriptome tree. Maximum‐likelihood phylogeny of 15 nudibranch transcriptomes constructed with 1,126 concatenated amino acid alignments. Members of the Fast 5 dataset appear in bold. Photographs are provided for each chromodorid specimen chosen for transcriptome sequencing (see Table 1), with the exception of *C. westraliensis* where the photograph represents a specimen chosen for exon capture (WAMS92252)

Post hoc comparisons showed that RNA extractions significantly differed in concentration depending on extraction protocol (one‐tailed *t*‐test, *p* = .01), with an average of 177.9 ng/μl using the Trizol protocol (*N* = 7) (range = 22.7–311.1) and 50.6 ng/μl using the Qiagen RNeasy protocol (*N* = 6) (range = 15.2–81.8). The total number of raw sequence reads ranged from 32.8 to 47.5 million per sample, with an average of 39.9 million, and the total number of transcripts ranged from 88,185 to 180,070, with an average of 137,528 (Table 1). The mean length of assembled transcripts ranged from 670 to 954 bp, with an average of 787 bp. The length of assembled transcripts significantly differed between samples that were extracted with the Trizol and Qiagen protocols (one‐tailed *t* test, *p* = .04), with an average of 823 bp in the former and 757 bp in the latter. There was no significant correlation between number of reads and mean transcript length (*p* = .09). A maximum‐likelihood (ML) analysis of all fifteen nudibranch transcriptomes is presented in Figure 2.

**Table 1:** Transcriptome data

Registration number	BioSample accession	Species name	RNA extraction protocol	RNA conc. (ng/μl)	Number of raw reads (millions)	Number of transcripts	Mean length of assembled transcripts (bp)
WAMS103009	SAMN15762361	*Verconia norba*	Trizol	311.1	34.2	119,946	670
WAMS103167	SAMN15762358	*C. magnifica*	Trizol	297.9	35.9	150,167	770
WAMS103020	SAMN15762362	*Ardeadoris egretta*	Trizol	254.2	33.5	108,588	761
WAMS103064	SAMN15762359	*Mexichromis festiva*	Trizol	156.6	33.1	156,225	779
SIO‐BIC‐M16703	SAMN15762364	*Felimida macfarlandi*	Trizol	122.0	33.3	114,561	797
WAMS70417	SAMN15762355	*Hypselodoris saintvincentius*	Qiagen	81.8	46.3	152,829	839
WAMS103163	SAMN15762360	*Ceratosoma tenue*	Trizol	80.7	32.8	149,243	761
WAMS70571	SAMN15762352	*Verconia verconis*	Qiagen	67.2	45.6	126,179	750
WAMS16470	SAMN15762357	*C. westraliensis*	Qiagen	59.4	45.9	180,070	954
WAMS92135	SAMN15762356	*Goniobranchus fidelis*	Qiagen	41.0	46.5	176,883	806
WAMS92136	SAMN15762353	*Goniobranchus coi*	Qiagen	38.8	47.5	155,871	854
WAMS72483	SAMN15762363	*Actinocyclus verrucosus*	Trizol	22.7	37.4	88,185	760
WAMS92138	SAMN15762354	*Doriprismatica atromarginata*	Qiagen	15.2	46.2	109,120	735
SIO‐BIC M12127	**SAMN02870751**	***Doris kerguelenensis***			**14.8**	**54,532**	**575**
YPM IZ 047929	**SAMN02870748**	***Prodoris clavigera***			**12.9**	**76,393**	**622**

Note

Museum registration, SRA accession, species name, extraction protocol, RNA concentration, number of raw reads, number of transcripts, and mean length of assembled transcripts. Data retrieved from GenBank are presented in bold and *Chromodoris* records appear underlined. Specimens are ordered from highest to lowest RNA concentration.

A total of 1,126 homologous genes were recovered with an amino acid search of all fifteen nudibranch transcriptomes (all nudibranchs in this study) while 4,065 genes were recovered with a nucleotide search of five closely related transcriptomes (Fast 5) (Figure 1). The total number of loci recovered from each search, including those extracted from Teasdale et al. (2016), is represented in Figure 1b. After genes were filtered for phylogenetic informativeness, the number of genes and exons (>120 bp) targeted for sequence capture totaled 1,774 and 2,925, respectively. The targeted genes ranged in length from 120 to 4,163 bp. The average number of targeted exons per gene was 1.78. Pairwise distances of gene alignments from *C. westraliensis* and *G. coi* and each Agalma search are presented in Figure 1c.

The total volume of DNA for exon capture ranged from 10 to 180 µl, and the concentration ranged from 6.84 to 500 ng/µl. A total of three samples originated from pooled extractions; the original concentrations of the extractions ranged from 0.27 to 7.88 ng/µl prior to pooling. Capture efficiency varied between species, with the number of genes with contigs ranging from 1,061 (60%) in the outgroup *D. atromarginata* to 1,667 (94%) in *C*. cf. *lochi* FPV (UF368685). *Chromodoris aspersa* (WAMS67676) had the lowest number of genes with contigs (1,456, 82.1%) within *Chromodoris*. The number of reads and genes mapped per sample is available in Table 2. Percentage of reads on target ranged from 41.3% in the outgroup *G. fidelis* to 78.3% in *C. kuiteri* (WAMS103139), with an average of 60% for all *Chromodoris*. A total of 144 genes were removed that did not enrich, or enriched poorly, likely due to intron sites that differed between *Chromodoris* and *Aplysia*. The dataset used for the phylogenetic analysis consisted of 1,630 genes (full‐gene dataset) from the combined Agalma searches and Teasdale et al. (2016) dataset, and each gene was aligned and trimmed with an average length of 347 bp post trimming. The final alignment was 566,204 bp in length, and the percentage of parsimony‐informative sites was 9.6% across all *Chromodoris*. The majority of genes sequenced from baits (*N* = 1,085) were derived from loci in the Fast 5 dataset.

**Table 2:** Target capture data

Registration number	Species name	Collection locality	DNA conc. (ng/µl)	Mean quality score	Reads mapped (millions)	Reads on target	Genes mapped
CASIZ192287	*C*. aff. *aspersa*	Saudi Arabia	17.2	35.22	1.23	0.607	1,665
CASIZ192279	*C*. aff. *aspersa*	Saudi Arabia	17.6	34.94	0.89	0.564	1,661
CASIZ176754	*C*. aff. *elisabethina* A	Malaysia	18.0	35.4	1.94	0.682	1,703
UF305137	*C*. aff. *elisabethina* B	Dededo, Guam	16.8	35.26	1.34	0.661	1,696
CASIZ121007	*C*. aff. *elisabethina* B	Dededo, Guam	>500	35.29	1.62	0.735	1,705
CASIZ181260	*C*. aff. *mandapamensis*	Batangas, Philippines	9.12	35.33	1.79	0.657	1,702
WAMS96283	*C*. aff. *striatella* WA A	Montebello Islands, WA, Australia	6.84	35.02	1.27	0.595	1,695
WAMS99380	*C*. aff. *striatella* WA B	Port Hedland, WA, Australia	16.5	34.91	1.19	0.621	1,703
WAMS35107	*C*. aff. *striatella* WA B	Port Hedland, WA, Australia	17.7	35.11	1.63	0.608	1,699
WAMS56055	*C*. aff. *willani*	Ningaloo, WA, Australia	18.3	35.27	2.91	0.644	1,711
CASIZ177260	*C*. aff. *willani*	Batangas, Philippines	20.4	35.27	2.09	0.683	1,710
CASIZ194439	*C. africana*	Madagascar	21.2	35.1	1.29	0.627	1,695
CASIZ194460	*C. africana*	Madagascar	14.0	35.26	2.64	0.663	1,706
CASIZ204143	*C. annae*	Batangas, Philippines	18.8	35.21	2.14	0.684	1,708
WAMS67522	*C. annae*	Sulawesi, Indonesia	17.3	35.12	1.51	0.654	1,701
CASIZ191422	*C. aspersa*	Madang, Papua New Guinea	38.9	34.75	0.83	0.484	1,658
WAMS67676	*C. aspersa*	Kimberley, WA, Australia	17.1	34.6	0.60	0.472	1,657
WAMS103006	*C. burni*	Mudjimba Island, QLD, Australia	12.1	34.82	2.06	0.598	1,711
WAMS103007	*C. burni*	Mudjimba Island, QLD, Australia	17.4	35.02	1.35	0.588	1,704
WAMS70791	*C*. cf. *africana*	Aliwal Shoal, South Africa	8.30	35.36	1.59	0.729	1,707
CASIZ177426	*C*. cf. *burni*	Batangas, Philippines	17.9	35.27	1.29	0.641	1,705
CASIZ177428	*C*. cf. *burni*	Batangas, Philippines	18.9	35.25	1.51	0.644	1,705
WAMS67532	*C*. cf. *dianae*	Sulawesi, Indonesia	23.2	35.23	1.57	0.664	1,704
WAMS67536	*C*. cf. *dianae*	Sulawesi, Indonesia	16.3	35.24	1.81	0.578	1,707
SBMNH89038	*C*. cf. *lochi* FP	French Polynesia	16.8	34.97	2.44	0.639	1,705
UF400236	*C*. cf. *lochi* FP	French Polynesia	14.1	35.24	1.87	0.629	1,710
WAMS67573	*C*. cf. *lochi* FPV	French Polynesia	11.1	35.11	2.08	0.659	1,718
UF368685	*C*. cf. *lochi* FPV	Samna Province, Vanuatu	18.2	35.34	0.76	0.664	1,714
WAMS103008	*C*. cf. *striatella* QLD	Mooloolaba, QLD, Australia	55.0	34.93	1.45	0.573	1,704
CASIZ177676	*C*. cf. *striatella* spotted	Batangas, Philippines	18.8	35.1	1.67	0.649	1,706
WAMS35075	***C. colemani* (*westraliensis* mimic)**	Rottnest Island, WA, Australia	22.8	35	1.38	0.519	1,710
CASIZ177266	*C. colemani*	Batangas, Philippines	19.1	35.11	1.24	0.534	1,715
WAMS103005	*C. colemani* (*C. col* x *burni*)	Mudjimba Island, QLD, Australia	2.80	35.15	1.77	0.624	1,712
WAMS67533	*C. dianae*	Sulawesi, Indonesia	16.4	35.22	1.25	0.562	1,711
CASIZ177242	*C. dianae*	Batangas, Philippines	19.4	35.16	1.04	0.563	1,708
WAMS67521	*C. elisabethina* (*C. elisabethina* x *annae*)	Sulawesi, Indonesia	22.0	35.35	0.96	0.520	1,713
WAMS67542	*C. elisabethina*	Mooloolaba, QLD, Australia	20.8	35.09	1.27	0.545	1,712
CASIZ194415	*C. hamiltoni*	Madagascar	13.6	35.29	1.61	0.725	1,699
CASIZ194587	*C. hamiltoni*	Madagascar	19.2	35.26	1.53	0.538	1,718
WAMS67657	*C. joshi* (*C. joshi* x *magnifica*)	Ningaloo, WA, Australia	20.4	35.29	0.72	0.531	1,706
CASIZ217220	*C. joshi*	Batangas, Philippines	20.8	35.02	1.80	0.625	1,698
WAMS103139	*C. kuiteri*	Mooloolaba, QLD, Australia	14.8	35.59	1.49	0.783	1,707
WAMS67546	*C. kuiteri*	Mooloolaba, QLD, Australia	14.0	35.24	1.39	0.659	1,710
UF310537	*C. lineolata*	Ulebeschel Island, Palau	20.8	35.14	1.19	0.654	1,699
WAMS67527	*C. lineolata*	Lizard Island, QLD, Australia	17.9	35.5	2.19	0.685	1,721
WAMS67566	*C. lochi*	Sulawesi, Indonesia	19.9	35.23	1.75	0.638	1,705
CASIZ182290	*C. lochi*	Romblon, Philippines	20.4	35.29	1.82	0.590	1,711
WAMS92170	*C. magnifica*	Muiron Islands, WA, Australia	17.3	34.97	1.07	0.598	1,702
CASIZ204796	*C. magnifica* (*C. magnifica* x meso)	Oriental Mindoro, Philippines	20.0	35.29	1.36	0.651	1,702
CASIZ194453	*C. mandapamensis*	Madagascar	17.0	35.33	1.25	0.673	1,700
CASIZ182807	*C. michaeli*	Batangas, Philippines	21.2	35.23	1.40	0.633	1,702
MMRBK457	*C. orientalis*	North Gyeongsang, South Korea	20.4	35.34	2.36	0.683	1,714
CASIZ192286	*C. quadricolor*	Saudi Arabia	14.5	35.31	2.29	0.681	1,710
WAMS67596	*C*. sp. IP	Sulawesi, Indonesia	19.5	35.12	1.04	0.493	1,720
CASIZ204798	*C*. sp. meso	Oriental Mindoro, Philippines	20.0	35.2	1.35	0.480	1,718
CASIZ204797	*C*. sp. meso	Oriental Mindoro, Philippines	19.9	34.99	1.34	0.500	1,717
CASIZ192505	*C*. sp. SA	Saudi Arabia	18.0	35.37	1.32	0.500	1,711
WAMS99382	*C. striatella*	Port Hedland, WA, Australia	20.8	35.05	1.66	0.522	1,714
AMC415149C	*C. striatella*	Shoalwater Bay, QLD, Australia	19.7	35.39	1.17	0.510	1,718
WAMS103147	*C. strigata*	Mooloolaba, QLD, Australia	17.1	35.11	1.09	0.464	1,706
CASIZ199453	*C. strigata*	Occidental Mindoro, Philippines	14.1	35.27	2.62	0.664	1,707
WAMS56037	*C. westraliensis*	Ningaloo, WA, Australia	17.2	35.2	1.09	0.481	1,711
WAMS92252	*C. westraliensis*	Muiron Islands, WA, Australia	18.0	34.9	1.36	0.564	1,701
CASIZ202316	*C. willani*	Batangas, Philippines	17.2	35.04	2.52	0.608	1,712
WAMS67603	*C. willani*	Sulawesi, Indonesia	18.0	35.09	2.36	0.655	1,708
WAMS92135	*Goniobranchus fidelis*	Exmouth Gulf, WA, Australia	19.8	34.28	0.44	0.413	1,376
WAMS92136	*Goniobranchus coi*	Exmouth Gulf, WA, Australia	13.9	34.31	0.44	0.416	1,381
WAMS92138	*Doriprismatica atromarginata*	Exmouth Gulf, WA, Australia	>500	35.05	1.16	0.561	1,407
WAMS103020	A*rdeadoris egretta*	Mooloolaba, QLD, Australia	23.2	34.71	0.77	0.425	1,414

Note

Museum registration, species name, collection locality, DNA concentration, mean quality score, number of reads mapped, proportion of reads on target, and number of genes mapped. Data generated by the Hybpiper pipeline are highlighted in blue. Mimics appear in bold and individuals with mitonuclear discordance appear underlined, with names in brackets representing discordant molecular signals. Species names derived from Layton et al. (2018).

### 3.2 Exploring mitonuclear discordance in *Chromodoris* phylogeny

**Figure 3:**
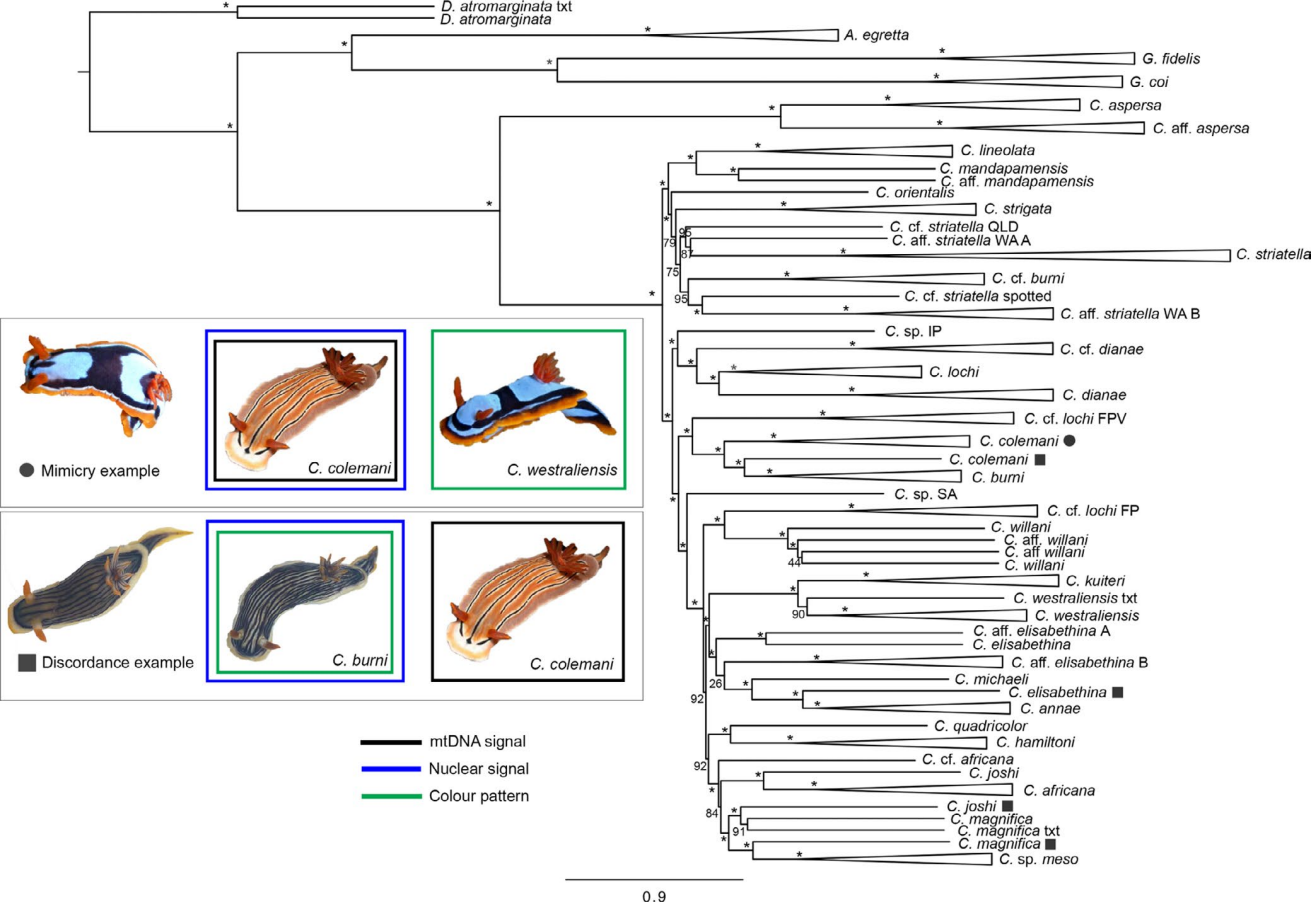
Mimicry and mitonuclear discordance in nudibranchs. ASTRAL summary coalescent tree of 1,630 genes with 100 bootstrap replicates. Bootstrap support is indicated at each node, with an asterisk marking nodes with 100% support. Triangles represent collapsed clades. Individuals with mitonuclear discordance are marked with a square, and mimics are marked with a circle (see inset). Names derived from Layton et al.(2018), and “txt” denotes a transcriptome sample. Inset: cases of mimicry and mitonuclear discordance from this study. Colored squares around each image match the legend provided, where identification derives from either mitochondrial signal, nuclear signal or color pattern. *C. colemani* (Photographer: Gary Cobb) and *Cwestraliensis* (Photographer: Bruce Potter) images derived from the Sea Slug Forum

The full‐gene ASTRAL phylogeny recovered most species‐level entities and sister–species relationships with high support (Figure 3). The number of species recovered in both the ASTRAL and concatenated ML analyses was largely congruent with a recent mtDNA analysis (Layton et al., 2018), with the exception of four individuals that showed mitonuclear discordance (Figure 3). In all four cases, the nuclear signal and color pattern were concordant, but the mitochondrial signal was that of another species, including two individuals that were previously identified as mimics in Layton et al. (2018). This includes the “burni” morph of *C. colemani* (*C. colemani* × *burni*), which had the mitogenome of *C. colemani* but with the nuclear signal and color pattern of *C. burni*, and the “magnifica” morph of *C. joshi* (*C. joshi* × *magnifica*), which had the mitogenome of *C. joshi* but with the nuclear signal and color pattern of *C. magnifica*. Additionally, a specimen that was previously identified as *C. magnifica* (*C. magnifica* × meso) had the mitogenome of *C. magnifica* but with the nuclear signal and color pattern of a new mesophotic reef species (*C*. sp. meso), and a specimen that was previously identified as *C. elisabethina* had the mitogenome of *C. elisabethina* but with the nuclear signal and color pattern of *C. annae* (*C. elisabethina* × *annae*). In contrast, one other mimic identified in Layton et al. (2018), the “westraliensis” morph of *C. colemani*, had concordant molecular signals.

**Figure 4:**
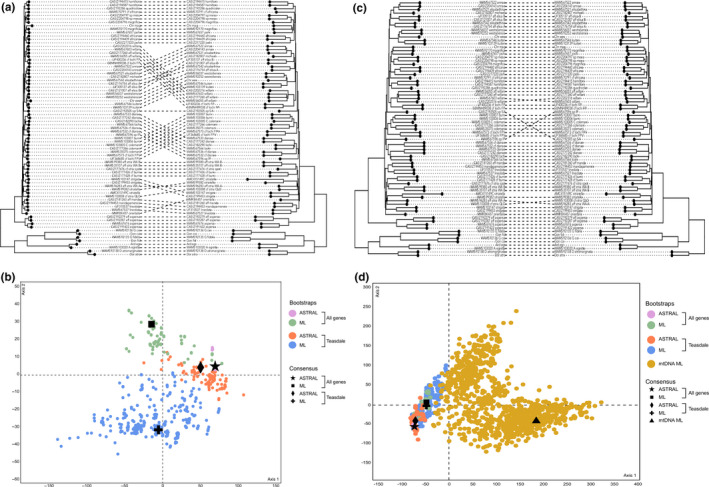
Exploring topological variability. (a) Concatenated ML tree (left) and ASTRAL summary coalescent tree (right) of the full dataset (*N* = 1,630). (b) Analysing tree landscapes from different datasets (All genes, Teasdale) and different tree‐building methods (concatenated ML, ASTRAL). (c) ASTRAL summary coalescent tree of the full dataset (*N* = 1,630) (left) and ASTRAL summary coalescent tree of the Teasdale dataset (*N* = 149) (right). (d) Treespace analysis from part B including mtDNA data that derives from Layton et al. (2018). Colors represent sets of bootstrap trees generated with different methods and/or data and symbols represent consensus trees

Despite the recovery of similar species‐level entities in the full‐gene ASTRAL and ML phylogenies, some topological differences occurred between the two analyses (Figure 4a). The position of the *C*. cf. *lochi* FP + *C*. aff. *willani* + *C. willani* and the *C. hamiltoni* + *C. quadricolor* clades was reversed in the ASTRAL and ML analyses. Moreover, *C*. cf. *burni* was recovered as sister to the *C*. cf. *striatella* spotted + *C*. aff. *striatella* WA B clade in the ASTRAL analysis, but as sister to *C. strigata* in the ML analysis. Lastly, the *C. magnifica* x meso + *C*. sp. meso clade was recovered as sister to the *C. magnifica* + *C. joshi* clade in the ASTRAL analysis, but as sister to *C*. cf. *africana* in the ML analysis. In the ML analysis, all three spotted species (*C*. aff. *aspersa*, *C. aspersa*, *C. orientalis*) were recovered as sister to the rest of the “striped” *Chromodoris*, but the position of *C. orientalis* differed in the ASTRAL phylogeny. Discordant parts of the phylogenies were still strongly supported, with bootstrap values >70.

A Treespace analysis recovered two tree islands; one comprised the full‐gene ASTRAL, full‐gene ML, and the Teasdale gene ASTRAL analyses, and a second contained only the Teasdale ML analysis (Figure 4b). The lowest variance was observed in the full‐gene ASTRAL analysis while the highest variance was observed in the Teasdale gene ML analysis (i.e., this cluster represented the most spread). When comparing across both tree‐building methods (ASTRAL and ML) and both datasets (full‐gene and Teasdale), the ML phylogenies appeared the least congruent (Figure 4b) while the ASTRAL phylogenies appeared the most congruent (Figure 4b). ASTRAL phylogenies of the full and Teasdale gene datasets were also largely congruent (Figure 4c). When mtDNA data were incorporated into the Treespace analysis, two distinct tree islands were produced (Figure 4d). One island contained the mtDNA data, which showed large variance, and a second, more tightly clustered island contained only those trees from exon data.

### 3.3 Assessing introgression among closely related species

After pruning for linkage disequilibrium, 3,811 SNPs were retained from a total 66,247. For individuals with mitonuclear discordance, the heterozygosity of fixed alleles ranged from a minimum of 6.03% (WAMS67521) to a maximum of 12.50% (CASIZ204796) and the number of SNPs that were fixed in the parental lineage but for which introgressed individuals were heterozygous ranged from a minimum of 129 in the *C. colemani* × *burni* individual (WAMS103005) to a maximum of 314 SNPs in the *C. joshi* × *magnifica* individual (WAMS67657). The expected percentage of heterozygosity for F1 and F2 hybrids would be 100% and 50%, respectively. Further, hybridization detection analysis in HyDe revealed 80 significant results, indicating widespread introgression among closely related species, but only two of four individuals with mitonculear discordance (*C. elisabethina* × *annae* and *C. colemani* × *burni*) were recovered as putative hybrids (Table 3).

**Table 3:** Introgression results

Parent 1	Hybrid	Parent 2	*Z*‐score	*p*‐Value	Gamma
*C*. aff. *elisabethina* A	*C. colemani*	*C*. cf. *striatella* QLD	4.86	5.87E−07	0.615
*C*. aff. *elisabethina* A	*C. willani*	*C*. cf. *lochi* FP	4.79	8.55E−07	0.301
*C*. aff. *elisabethina* B	***C. elisa*. × *annae***	*C. elisabethina*	4.86	5.88E−07	0.559
*C*. aff. *striatella* WA A	*C*. cf. *africana*	*C. elisabethina*	5.20	9.77E−08	0.318
*C*. aff. *striatella* WA A	*C*. cf. *africana*	*C. hamiltoni*	5.87	2.17E−09	0.307
*C*. aff. *striatella* WA A	*C*. cf. *africana*	*C. joshi* × *mag*.	4.83	6.76E−07	0.241
*C*. aff. *striatella* WA A	*C. colemani*	*C. hamiltoni*	4.94	3.93E−07	0.493
*C*. aff. *striatella* WA A	*C. quadricolor*	*C. hamiltoni*	5.04	2.33E−07	0.228
*C*. aff. *striatella* WA B	*C*. cf. *africana*	*C. hamiltoni*	4.82	7.25E−07	0.214
*C*. aff. *striatella* WA B	*C*. cf. *lochi* FPV	*C. elisabethina*	4.97	3.36E−07	0.426
*C*. aff. *striatella* WA B	*C*. cf. *lochi* FPV	*C. hamiltoni*	5.17	1.17E−07	0.423
*C*. aff. *striatella* WA B	*C*. cf. *lochi* FPV	*C. michaeli*	4.87	5.72E−07	0.350
*C*. aff. *striatella* WA B	*C*. cf. *striatella* spotted	*C. *aff. *mandapamensis*	4.99	3.03E−07	0.732
*C*. aff. *striatella* WA B	*C*. cf. *striatella* spotted	*C*. cf. *burni*	4.79	8.15E−07	0.649
*C*. aff. *striatella* WA B	*C*. cf. *striatella* spotted	*C. lineolata*	5.43	2.85E−08	0.658
*C*. aff. *striatella* WA B	*C*. cf. *striatella* spotted	*C. elisa*. × *annae*	5.04	2.38E−07	0.754
*C*. aff. *striatella* WA B	*C. colemani*	*C. elisabethina*	5.10	1.74E−07	0.360
*C*. aff. *striatella* WA B	*C. colemani*	*C. hamiltoni*	5.40	3.33E−08	0.377
*C*. aff. *striatella* WA B	*C. colemani*	*C. michaeli*	4.99	3.07E−07	0.329
*C*. aff. *willani*	*C. annae*	*C. elisabethina*	5.10	1.67E−07	0.485
*C*. aff. *willani*	*C*. cf. *africana*	*C. hamiltoni*	4.96	3.49E−07	0.518
*C*. aff. *willani*	*C. colemani*	*C*. cf. *striatella* QLD	4.74	1.09E−06	0.634
*C*. aff. *willani*	***C. elisa*. × *annae***	*C. elisabethina*	4.95	3.81E−07	0.417
*C. africana*	*C. willani*	*C*. cf*. lochi* FP	4.96	3.44E−07	0.332
*C. annae*	*C*. aff. *willani*	*C*. cf. *lochi* FP	4.88	5.38E−07	0.462
*C. annae*	*C. willani*	*C*. cf. *lochi* FP	5.33	4.98E−08	0.422
*C*. cf. *burni*	*C*. cf. *striatella* spotted	*C. lineolata*	6.06	7.04E−10	0.522
*C*. cf. *burni*	*C. colemani*	*C. elisabethina*	4.75	1.03E−06	0.365
*C*. cf. *lochi* FP	*C*. aff. *willani*	*C. magnifica*	4.90	4.78E−07	0.583
*C*. cf. *lochi* FP	*C*. aff. *willani*	*C. michaeli*	4.73	1.14E−06	0.665
*C*. cf. *lochi* FP	*C*. aff. *willani*	*C. elisa*. × *annae*	5.25	7.47E−08	0.602
*C*. cf. *lochi* FP	*C*. aff. *willani*	*C. joshi* × *mag*.	5.42	3.06E−08	0.611
*C*. cf. *lochi* FP	*C*. aff. *willani*	*C. mag*. × meso	5.84	2.65E−09	0.512
*C*. cf. *lochi* FP	*C*. cf. *africana*	*C*. sp. SA	5.12	1.49E−07	0.498
*C*. cf. *lochi* FP	*C. willani*	*C. joshi*	5.16	1.26E−07	0.650
*C*. cf. *lochi* FP	*C. willani*	*C. magnifica*	5.46	2.41E−08	0.610
*C*. cf. *lochi* FP	*C. willani*	*C. michaeli*	5.91	1.76E−09	0.650
*C*. cf. *lochi* FP	*C. willani*	*C. elisa*. × *annae*	6.14	4.02E−10	0.605
*C*. cf. *lochi* FP	*C. willani*	*C. joshi* × *mag*.	5.67	7.04E−09	0.651
*C*. cf. *lochi* FP	*C. willani*	*C. mag*. × meso	6.62	1.75E−11	0.544
*C*. cf. *striatella* QLD	*C. colemani*	*C. michaeli*	5.35	4.49E−08	0.431
*C*. cf. *striatella* QLD	*C. colemani*	*C. elisa*. × *annae*	5.20	1.02E−07	0.344
*C*. cf. *striatella* QLD	***C. col*. × *burni***	*C. michaeli*	5.31	5.50E−08	0.447
*C*. cf. *striatella* spotted	*C. annae*	*C. hamiltoni*	4.83	6.83E−07	0.315
*C*. cf. *striatella* spotted	*C*. cf. *africana*	*C. hamiltoni*	5.63	9.06E−09	0.273
*C*. cf. *striatella* spotted	*C. colemani*	*C. hamiltoni*	5.06	2.08E−07	0.475
*C*. cf. *striatella* spotted	*C*. sp. meso	*C. hamiltoni*	5.52	1.68E−08	0.344
*C. dianae*	*C*. cf. *africana*	*C. lineolata*	4.92	4.44E−07	0.586
*C. elisabethina*	*C. colemani*	*C*. cf. *striatella* QLD	5.20	9.85E−08	0.535
*C. hamiltoni*	*C. africana*	*C. mag*. × meso	4.98	3.11E−07	0.475
*C. hamiltoni*	*C*. cf. *africana*	*C. dianae*	4.80	8.01E−07	0.753
*C. hamiltoni*	*C*. cf. *africana*	*C*. sp. SA	5.19	1.04E−07	0.609
*C. hamiltoni*	*C*. cf. *africana*	*C. mag*. × meso	5.25	7.50E−08	0.338
*C. hamiltoni*	*C. colemani*	*C*. cf. *striatella* QLD	5.39	3.60E−08	0.518
*C. hamiltoni*	*C. quadricolor*	*C*. sp. SA	4.89	4.94E−07	0.711
*C. joshi*	*C*. cf. *africana*	*C. quadricolor*	5.03	2.48E−07	0.518
*C. joshi*	*C. colemani*	*C*. cf. *striatella* QLD	5.05	2.19E−07	0.587
*C. lineolata*	*C*. cf. *striatella* spotted	*C*. cf. *striatella* QLD	5.79	3.43E−09	0.593
*C. mandapamensis*	*C*. cf. *lochi* FPV	*C. kuiteri*	4.91	4.46E−07	0.481
*C. mandapamensis*	*C*. cf. *lochi* FPV	*C. elisa*. × *annae*	5.02	2.55E−07	0.484
*C. mandapamensis*	*C*. cf. *lochi* FPV	*C. joshi* × *mag*.	4.79	8.40E−07	0.575
*C. mandapamensis*	*C. strigata*	*C*. aff. *mandapamensis*	7.17	3.82E−13	0.615
*C. orientalis*	*C*. cf. *striatella* spotted	*C. lineolata*	4.85	6.16E−07	0.484
*C. quadricolor*	*C*. cf. *africana*	*C. magnifica*	4.84	6.44E−07	0.468
*C. quadricolor*	*C*. cf. *africana*	*C. mag*. × meso	5.54	1.51E−08	0.432
*C*. sp. meso	*C*. aff. *willani*	*C*. cf. *lochi* FP	5.25	7.55E−08	0.525
*C*. sp. meso	*C*. cf. *africana*	*C. quadricolor*	5.36	4.10E−08	0.617
*C*. sp. meso	*C. willani*	*C*. cf. *lochi* FP	6.03	8.28E−10	0.474
*C*. sp. SA	*C*. cf. *africana*	*C. michaeli*	5.35	4.52E−08	0.434
*C*. sp. SA	*C*. cf. *africana*	*C. mag*. × meso	4.97	3.33E−07	0.261
*C. striatella*	*C*. cf. *africana*	*C. hamiltoni*	4.80	8.00E−07	0.237
*C. strigata*	*C. *aff*. mandapamensis*	*C. lineolata*	5.38	3.79E−08	0.519
*C. westraliensis*	*C*. cf. *lochi* FPV	*C. mandapamensis*	4.79	8.25E−07	0.479
*C. willani*	*C. annae*	*C. elisabethina*	4.96	3.60E−07	0.493
*C. willani*	*C*. cf. *africana*	*C. hamiltoni*	4.72	1.17E−06	0.531
*C. willani*	***C. elisa*. × *annae***	*C. elisabethina*	5.11	1.61E−07	0.449
*C. joshi* × *mag*	*C*. cf. *africana*	*C. quadricolor*	5.86	2.34E−09	0.477
*C. joshi* × *mag*	*C*. cf. *africana*	*C*. sp. SA	5.18	1.10E−07	0.685
*C. joshi* × *mag*	*C. colemani*	*C*. cf. *striatella* QLD	5.16	1.22E−07	0.597

Note

Significant results from hybridization detection analysis in HyDe (Blischak et al., 2018), with corresponding *Z*‐scores, *p*‐values and Gamma values. Mimetic species with mitonuclear discordance that appear as hybrids are highlighted in bold and have been renamed with the abbreviated form of their putative parental lineages.

## 4 DISCUSSION

### 4.1 An informative bait set for *Chromodoris* nudibranchs

This study has established an informative bait set to improve phylogenetic resolution in a group of recently radiated nudibranchs, where post‐glacial expansion of allopatric lineages into new regions and increased ecological opportunity has likely promoted rapid divergence (i.e., Losos, 2010). This study employed two different search methods in Agalma in order to identify a robust set of phylogenetically informative homologous genes. The two search methods used here (amino acid and nucleotide) returned different sets of genes and this is likely because (a) *Chromodoris* has only recently radiated and thus many genes would be invariant at the amino acid level and would have been discarded in the Agalma filtering steps and (b) because translation only occurs in the amino acid search so genes with frameshift errors in the transcriptome assemblies would have been discarded in the former (Teasdale et al., 2016). It is unlikely that a nucleotide search returned more genes due to incorrect identification of paralogs as homologs because (a) a paralogy‐pruning step in the Agalma Phylogeny pipeline ensures that mostly orthologous genes are included in subsequent matrix construction, and (b) the Hybpiper analysis did not produce any paralog warnings, meaning that there were no cases of multiple contigs assembled de novo from the capture data mapping to >85% of the same gene target.

We also included a set of genes from Teasdale et al. (2016) that were expected to have broad phylogenetic utility, and we confirmed that a subset of these genes (*N* = 149) were informative for resolving sister–species relationships in *Chromodoris*. Although we originally predicted that the different gene sets would be informative at different scales of divergence, there was no difference in the distribution of evolutionary rates between the sets of genes.

### 4.2 Mitonuclear discordance and mimicry

This study employed phylogenomic data to test hypotheses of mimicry that derive from Layton et al. (2018). We uncovered mitonuclear discordance in two of these purported mimics, while in a third mimic, the mitochondrial and nuclear signals were concordant. Concordance among mitochondrial and nuclear signals renders this individual a true mimic, while discordance among molecular signals suggests that the others derive from introgression or mitochondrial capture. Only a few cases of mitonuclear discordance have been reported in marine molluscs to date (i.e., mussels, Quesada, Wenne, & Skibinski, 1999; Rawson & Hilbish, 1998; octopus, Amor et al., 2019), and our study adds the first known cases in nudbranchs where the mitochondrial signal is incongruent with both the nuclear signal and color pattern. Mitonuclear discordance can arise from incomplete lineage sorting (ILS), but it is likely that discordance in this study has arisen through introgression or mitochondrial capture, although population‐level sampling would help differentiate these patterns (Twyford & Ennos, 2012). First, Layton et al. (2018) demonstrate substantial mtDNA divergence among species that is largely supported with nuclear data in this study, suggesting that there is coalescence in these markers (Hinojosa et al., 2019). Secondly, these individuals exhibit morphologies that are strikingly similar to one of the putative parental lineages, which may support a scenario of asymmetric introgression (e.g., Wallace et al., 2011). Lastly, hybridization detection analysis indicates that two individuals with mitonuclear discordance are hybrids, although the parent species identified in this analysis do not match the expected parental lineages which might reflect ancestral hybridization or ILS. In any case, a lack of nuclear introgression in two other cases of discordance points toward a pattern of mitochondrial capture (Good, Vanderpool, Keeble, Bi, 2015). Introgression and mitochondrial capture are likely scenarios for this group given that they often occur sympatrically, are closely related, and have only recently radiated (e.g., Foltz, 1997; Mallet, Besansky, & Hahn, 2015). Although hybridization has been considered rare in marine invertebrates, previous work has shown evidence for introgression in several broadcast spawning invertebrates, including bivalves (e.g., Gardner, Skibinski, & Bajdik, 1993), ascidians (Nydam & Harrison, 2010; Nydam et al., 2017), and corals (Vollmer & Palumbi, 2002; Willis, van Oppen, Miller, Vollmer, & Ayre, 2006). Evidence for introgressive hybridization in *Chromodoris* is particularly intriguing given that they do not broadcast spawn but rather engage in copulation as simultaneous hermaphrodites. Rare instances of interspecific matings in Nudibranchia have been observed by divers, but because no study has uncovered hybrid individuals, it was generally assumed that postmating‐prezygotic reproductive isolation would prevent fertilization. This study is the first to uncover evidence for introgression and mitochondrial capture in nudibranchs, demonstrating that species delimitation relying solely on mitochondrial data is problematic for this group. Introgression has been crucial in the evolution of mimicry in other taxa (e.g., Pardo‐Diaz et al., 2012; *Heliconius* Genome Consortium, 2012), and thus future work will investigate the role of hybridization and mimicry in facilitating speciation in nudibranchs.

Color patterns have been employed for species delimitation in many groups of nudibranchs, but mimicry complicates these patterns. The nearly identical phenotypic match between mimetic *C. colemani* and the model *C. westraliensis* (differing only by a white line separating the black dorsum and orange margin in the former) in Western Australia is likely due to the lack of congenerics in the region, given that a mosaic color pattern would be expected in the presence of multiple co‐existing species (Akcali, Pérez‐Mendoza, Kikuchi, & Pfennig, 2019). The close match between mimic and model also confers a fitness advantage because predators learn this pattern more quickly (Mallet & Singer, 1987), although it remains unknown whether this mimicry is Müllerian or Batesian in nature. Although mimicry has been documented in tropical fish, with color patterns driving speciation in some groups (e.g., Puebla, Bermingham, Guichard, & Whiteman, 2007), less is known about mimicry in nudibranchs and in molluscs in general (but see Cheney et al., 2016; Winters et al., 2017; Winters, White, et al., 2018; Winters, Wilson, et al., 2018). A study by Padula et al. (2016) uncovered mimicry in another chromodorid genus (*Felimida*), but that study was based on mitochondrial data and further work is needed to determine whether this constitutes a “pure” case of mimicry or whether this pattern has arisen through introgression. Two mimics in this study were recovered as putative hybrids in a hybridization detection analysis, but additional genomic resources and population‐level sampling would advance our understanding of the evolutionary processes governing mimicry in this genus.

### 4.3 Topological incongruence and implications for *Chromodoris* phylogeny

This study reports incongruence between phylogenies generated with concatenated ML and ASTRAL summary coalescent methods, where the former produced phylogenies that were discordant between different datasets (i.e., sets of genes) and the latter produced highly concordant phylogenies consistent across different datasets. The highly variable phylogenies produced by the concatenated ML analyses suggest that this method underperforms for taxonomic groups that have recently diverged and where gene trees may be in conflict. In fact, Kubatko and Degnan (2007) demonstrated that concatenating loci when gene trees are in conflict can produce incorrect phylogenetic estimates, and therefore the choice of phylogenetic method is important for resolving relationships in recently radiated taxa. Bragg et al. (2018) found that concatenated ML and ASTRAL phylogenies of a rapid radiation of Australian skinks were largely congruent, contradicting results in this study. Nonetheless, incorporating data from a large number of loci means that species trees will often converge on a single topology (e.g., Blom, Bragg, Potter, & Moritz, 2016).

Some sister–species relationships differ between the full‐gene ASTRAL phylogeny in this study and the mitochondrial phylogeny from Layton et al. (2018), while some topologies remain the same in both mitochondrial and exon analyses. The position of spotted *C. orientalis* as sister to a clade containing striped *Chromodoris*, with spotted *C. aspersa* and *C*. aff. *aspersa* as sister to this larger clade, would suggest that spots are a plesiomorphic trait. However, this topology was only observed in the concatenated ML phylogeny and was not reflected in the ASTRAL analysis. The position of *C. orientalis*, *C*. aff. *aspersa*, and *C. aspersa* as sister to the striped *Chromodoris* is attractive in a parsimonious sense, given they are the only spotted members of this genus, and thus the absence of this pattern in the ASTRAL phylogeny is surprising. Future work could revisit this hypothesis by employing statistical binning methods for summary coalescent analysis, where gene trees are binned into sets with similar evolutionary histories and then combined into supertrees for ASTRAL (Bayzid, Mirarab, Boussau, & Warnow, 2014; Mirarab, Bayzid, Boussau, & Warnow, 2014). The evolution of spots could also be traced on a phylogeny of all chromodorid nudibranchs in order to determine the evolutionary status of this character in a broader context.

The exon capture approach employed in this study has significantly improved resolution of *Chromodoris* phylogeny and demonstrates, for the first time, mitonuclear discordance in nudibranchs likely originating from introgression or mitochondrial capture. This study also confirms a “pure” mimic in Western Australia, prompting additional investigation into the genomic basis of mimicry and warning colouration in these enigmatic animals.

## CONFLICT OF INTEREST

The authors declare no conflict of interest.

## AUTHOR CONTRIBUTIONS

**Kara K.S. Layton:** Conceptualization (equal); Data curation (equal); Formal analysis (equal); Funding acquisition (supporting); Investigation (equal); Methodology (equal); Project administration (lead); Resources (supporting); Validation (equal); Visualization (lead); Writing‐original draft (lead); Writing‐review & editing (equal). **Jose I. Carvajal:** Conceptualization (equal); Data curation (equal); Formal analysis (equal); Investigation (equal); Methodology (equal); Validation (equal); Visualization (supporting); Writing‐review & editing (equal). **Nerida G. Wilson:** Conceptualization (equal); Funding acquisition (lead); Investigation (equal); Methodology (supporting); Project administration (supporting); Resources (lead); Writing‐review & editing (equal).

## Data Availability

Assembled transcriptome sequences and phylogenetic datasets are available on Dryad: https://doi.org/10.5061/dryad.xd2547dfb. Raw reads are available on the Sequence Read Archive (SRA) from NCBI under the BioProject ID PRJNA655945, and accessions are provided in Table 1. Python scripts are available on Github: ignacio3437/agalmacap. All DNA extractions are vouchered at the Western Australian Museum.
